# An Editor

**Published:** 1857-10

**Authors:** 


### AN EDITOR

MUST be an industrious man.

He must have enlarged and liberal ideas.

He must be a man of general intelligence.

He must be a scholar.

He must possess great forbearance.

He must have a genial heart.

He must have clear, concise views.

He must be fearless as Cæsar, and firm as Gibraltar.

He must have no pride of consistency, but a perfect devotion to what is true.

He must have a steady aim to do right.

He must wage a ceaseless war against oppression and wrong-doing, in high places and low.

He must be endowed with great equanimity of temper.

He must have a vigorous digestion.

To be a useful editor, in the highest sense of the term, a man must possess all these traits. Such an one is a diamond of the first water

